# A Deep-Learning Approach for Foot-Type Classification Using Heterogeneous Pressure Data

**DOI:** 10.3390/s20164481

**Published:** 2020-08-11

**Authors:** Jonghyeok Chae, Young-Jin Kang, Yoojeong Noh

**Affiliations:** 1 School of Mechanical Engineering, Pusan National University (PNU), Busan 46290, Korea; jhchae92@pusan.ac.kr; 2 Research Institute of Mechanical Technology, Pusan National University (PNU), Busan 46290, Korea; zmanx@pusan.ac.kr

**Keywords:** heterogeneous pressure data, arch index, k-NN, fine-tuned VGG16, stacking ensemble

## Abstract

The human foot is easily deformed owing to the innate form of the foot or an incorrect walking posture. Foot deformations not only pose a threat to foot health but also cause fatigue and pain when walking; therefore, accurate diagnoses of foot deformations are required. However, the measurement of foot deformities requires specialized personnel, and the objectivity of the diagnosis may be insufficient for professional medical personnel to assess foot deformations. Thus, it is necessary to develop an objective foot deformation classification model. In this study, a model for classifying foot types is developed using image and numerical foot pressure data. Such heterogeneous data are used to generate a fine-tuned visual geometry group-16 (VGG16) and K−nearest neighbor (k-NN) models, respectively, and a stacking ensemble model is finally generated to improve accuracy and robustness by combining the two models. Through k-fold cross-validation, the accuracy and robustness of the proposed method have been verified by the mean and standard deviation of the f1 scores (0.9255 and 0.0042), which has superior performance compared to single models generated using only numerical or image data. Thus, the proposed model provides the objectivity of diagnosis for foot deformation, and can be used for analysis and design of foot healthcare products.

## 1. Introduction

The feet are composed of bones, muscles, and ligaments, and they enable continuous walking through organically connected movements with each component while making direct contact with the ground when walking. However, if there is a congenital problem with the foot shape or walking in an uncomfortable walking position such as in-toeing and out-toeing gait, the ground pressure will be concentrated on specific parts of the foot, resulting in permanent deformation of the foot, and may cause knee joint or back pain [1]. In particular, the feet are easily deformed due to the wrong walking posture, and the foot deformations not only pose a threat to foot health but also cause fatigue and pain while walking, and can also deform the spine; therefore, accurate diagnosis of foot deformations is required.

The deformation of the foot is mainly caused by a change in the arch of the foot, which comprises the medial longitudinal arch (MLA), lateral longitudinal arch (LLA), and anterior transverse arch (ATA). These arches have a concave shape, which helps to absorb shocks transmitted from the ground. Foot types can be classified as concave or flat feet depending on the height of the MLA [2]. In the case of concave feet, the deformation is caused by an abnormally high MLA, resulting in a rigid and shortened pericardial membrane, reducing the area of contact with the ground, and concentrating the load at foot support points. However, in the case of flat feet, the MLA is lost, causing the soles of the feet to deform in a flattened state. Flat feet weaken the shock absorption function and can cause diseases such as pericarditis, toe deformity, and ankle arthritis [3,4]. Thus, it is important to prevent foot deformation by performing periodic examinations as it may cause ankle and knee pain, lower limb disease, etc. [1,5].

One of the most important tests to check for foot deformation is to accurately measure the height of the MLA [1]. Accordingly, many methods, including radiographic examination, visual observation, and ink footprints, have been proposed for measuring the height of the arch and correlation of the actual measured MLA with foot deformation [6,7,8]. However, these methods either require expensive equipment or are difficult to use for accurate foot deformation measurements. Therefore, the arch index (AI) that uses the area of the midfoot is widely used because the area of the midfoot can be easily calculated. A recent study using the AI and ink footprints suggested that the AI and the heights of MLA have a high correlation coefficient of −0.7 [9], which can be useful in conducting biomechanical examinations [8,10,11,12]. The study using ink footprints is noninvasive and inexpensive, but it has some critical demerits as follows: (a) a discomfort to subjects due to coating the ink to soles, (b) a very complicated post-processing to calculate the AI due to all manual processes, (c) a dependency on the tester of the accuracy of results owing to the manual process. Recently, as a digital footprint measurement equipment, which uses pressure sensors to obtain electronic footprints and can automatically calculate the AI without the manual process, has been developed [13,14], data can be utilized to verify the measurement accuracy of the foot pressure being applied [9,14,15]. The modified arch index (MAI), which is obtained using digital data, was proposed based on the AI, which is most closely correlated with the height of the MLA, and the AI values of ink footprints were compared with those of electronic footprints [9,16,17,18]. Analyses that were carried out using the MAI in electronic footprints show the advantage of finding an abnormal AI by more evenly distributing data [16]. However, the problem is that the foot deformation results that are obtained for the same data may vary depending on expert opinion because the evaluation of foot deformation using these arch indices requires an expert’s subjective judgments. To mitigate these problems, it is necessary to develop an objective medical diagnosis evaluation method based on foot pressure data. 

Recent advances in machine-learning algorithms have enabled computers to better interpret highly complex data and achieve a very high accuracy in the analysis of data. In particular, deep learning, which is part of a family of machine-learning algorithms based on artificial neural networks (ANNs), has recently been actively researched in the medical field, as accurate diagnoses are possible through image data learning on complex body structures [19]. Deep-learning research in the medical field is mainly used to solve binary classification problems that distinguish normal and abnormal feet of the subjects based on supervised learning. There have been a growing number of cases in which deep learning is applied to medical data, to improve medical examination accuracy and treatment outcomes [19,20,21,22,23]. In particular, studies that incorporate foot pressure data and deep learning techniques to diagnose pediatric diseases have been reported recently [22,23]. Wang et al. [22] used density-based spatial clustering with noise (DBSCAN) and K-means clustering techniques to extract the walking characteristics of stroke patients and compared them with normal people in the weasel pressure data. In addition, Li et al. [23] attempted to extract the key areas needed to make customized shoes from the weasel pressure data of diabetics using convolutional neural networks (CNNs) and improved isometric MAPping (ISOMAP). As such, deep learning has been actively studied to improve accuracy in the medical diagnosis of various diseases, but no deep learning models have been developed for the purpose of diagnosing foot deformation using foot pressure data.

Previous studies suggested methods for classifying foot deformation through various measures, or developed an artificial intelligence model used for diagnosis of disease in the medical field. However, because medical experts should subjectively diagnose foot deformation types by footprint shapes or only the footprint area, diagnosis results for foot deformation may be inaccurate. Furthermore, they do not provide mathematical or numerical classification models for classifying foot deformation and therefore cannot be applied to analysis and design of smart foot healthcare products such as smart shoes and insoles. In addition, the artificial intelligence model developed for medical diagnosis purposes has not been customized for the diagnosis of foot deformation, so it may not be suitable as a classification model for foot deformation types. Therefore, in this study, an AI-based deep learning model was developed to classify foot deformation types into concave, normal, and flat feet based on image pressure data and numerical data. These heterogeneous data enrich information from which the classification can be derived [24], resulting in improved data quality and an increased accuracy of the classification model for foot deformation. For image data, fine-tuned visual geometry group-16 (VGG16) and InceptionV3 models, which are known as high-performance CNN-based models, were used. For numerical data, K−nearest neighbor (k−NN) and classifier and regression tree (CART), which are well-known as traditional classification algorithms [25,26,27], were used. The accuracy of the image-type-based models and numerical data-based models were compared using f1 score, and VGG16 and k−NN with the best performance were selected. Then, two heterogeneous models (VGG16 and k−NN) with high performance were combined as a stacking ensemble (SE) model, which learns the predicted values of the two models, thus improving the accuracy and robustness of each single model. Thus, the proposed method can improve the accuracy and robustness of diagnosis results and ensure objectivity, so it can be widely used in the analysis and design of products related to foot healthcare using foot deformation diagnosis results.

## 2. Data Preparation

This section describes the experimental method to collect the pressure of foot data, the calculation of AI to evaluate types of foot, and the pre-processing process of data. In general, raw data contain unnecessary information for learning, so data preprocessing should eliminate irrelevant information and update it with useful information. The amount of preprocessed data is less than the raw data, which not only reduces calculation time, but also increases the learning accuracy as only useful information is used for learning [23]. Therefore, the raw data need to be carefully refined before analysis. In this study, two types of data were collected: image data (pressure distribution) and numerical data (pressure value). According to the two types of data collected in this study, different pre-processing methods need to be used for deep learning. The method of data acquisition and calculation of the AI are explained in Section 2.1 and Section 2.2, respectively. The pre-processing steps for image data such as image rotation, resizing, and region of interest (ROI) are discussed in Section 2.3, and the feature extraction and selection for the numerical data are explained in Section 2.4. 

### 2.1. Data Acquisition

The subjects in this study were 96 adult men and women who voluntarily participated in the experiment through public recruitment. This study focuses on the development of artificial intelligence models for classifying foot deformation types, so the developed model only needs foot pressure and foot shape without any other personal information such as sex, height or weight. Therefore, without collecting such personal information, only the subjects’ foot pressure data were collected. Further, the purpose of this study is to develop foot healthcare products for the public, not for patients with walking-related diseases, and thus, we recruited subjects who had no walking-related diseases. All subjects in the study were informed about the purpose and nature of the pre-experimental test, signed a consent form, and participated in the experiment. The Institutional Review Board (IRB) Bioethics Committee of Pusan National University approved this study, and agreed in advance on all topics. 

**Figure 1:**
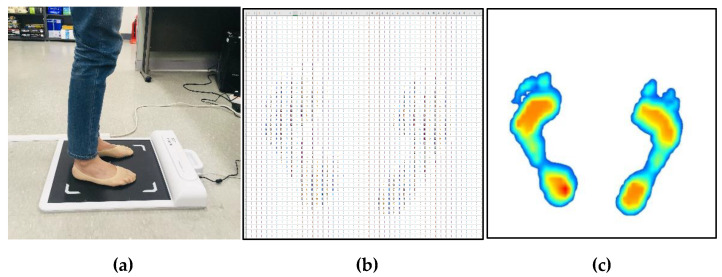
Experimental method and results. (**a**) Plantar pressure measurement, (**b**) numerical pressure data, (**c**) distribution of pressure image data.

Figure 1 shows the measurement method and data obtained from the measurement. All measurement procedures were conducted under the supervision of the research staff, and measurements were done with bare feet static standing using a G-hiwell’s Low Pile Pressure Measuring Device (GHF−550). During the data collection process, the method reported by Chu et al. [9] was used. Subjects were asked to stand in a standing position with both feet shoulder-width apart at the foot position indicated on the measurement device. In order to reduce the deviation of posture from the measurement, the subjects’ eyes were fixed towards the front wall, and the plantar pressure of the subjects was repeatedly measured two or three times on average until the pressure of the left and right feet was balanced in the standing position. The measurement device has a detection area of 400 mm × 400 mm, and consists of 2304 pressure sensors with 48 × 48 arrays. The pressure value data are measured in kPa, and the image data is an intensity color image of size 918 × 918, which was obtained by estimating the pressure distribution.

### 2.2. Calculation of Arch Index (Labeling)

**Figure 2:**
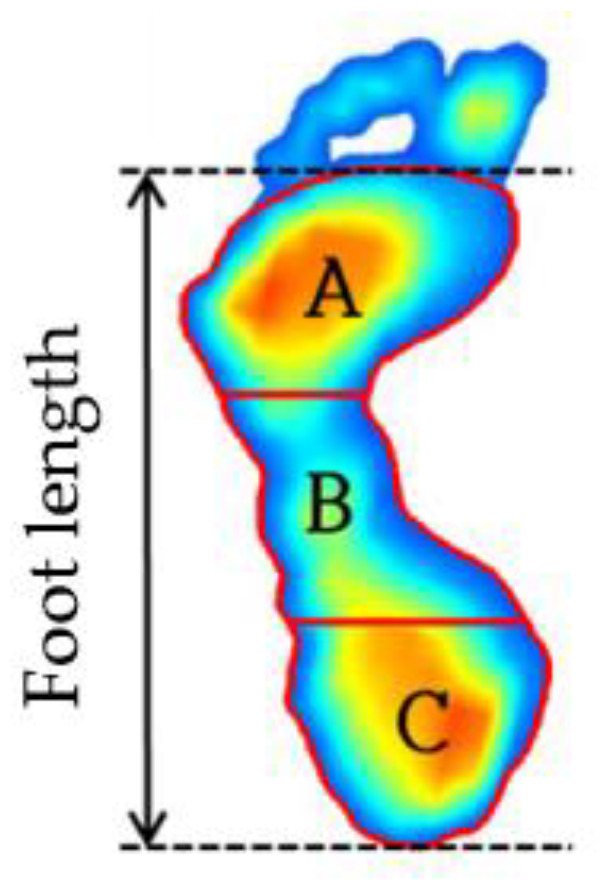
Arch index calculation method.

Supervised learning is the most commonly utilized machine-learning algorithm that is employed in medical imaging research. The supervised learning model acquires the relationship between input and output variables when the input and output data are given with labels. A supervised classification algorithm is a function that receives output data as a label value, and it categorizes input data into binary or multiple categories. In this study, the collected data are labeled as high (concave), mid (normal), and low (flat) depending on the height of the MLA [6,7,8,11]. The AI is the ratio of the area of the midfoot to the total area of the footprint, and is calculated by dividing the total length of the foot (the length between the heel end of the foot and the tip of the forefoot) into three equal parts, as shown in Figure 2 and Equation (1) [6]:

(1) $$AI=\frac{B}{A+B+C}$$

where *A*, *B*, and *C* are respectively the forefoot, midfoot, and hindfoot regions. As shown in the figure, the *AI* is calculated from toeless foot images, so this study also calculated the *AI* after removing the toe from the plantar image data during pre-processing of the data. The procedure for removing toe images will be discussed in detail in Section 2.3. 

**Figure 3:**
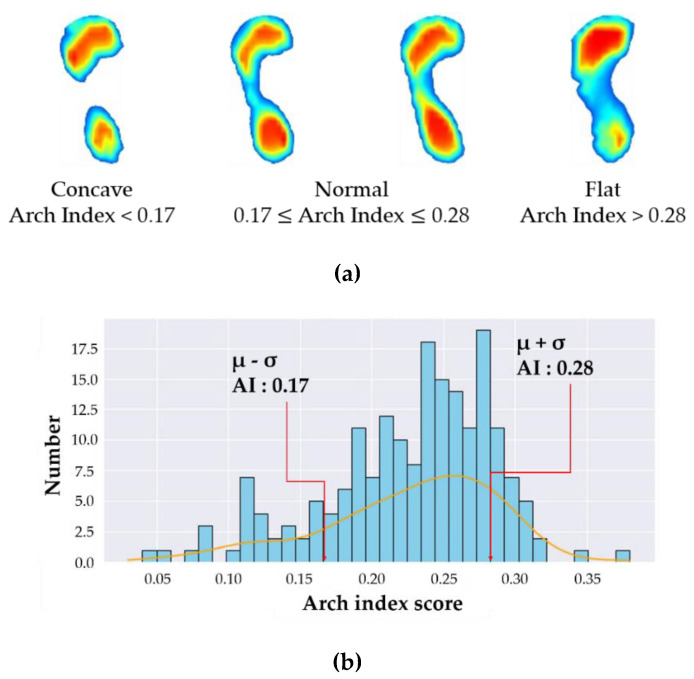
Labeled results and histogram of arch index data. (**a**) Arch index method and labeled image data, (**b**) Distribution of the data.

Two representative methods are employed to determine foot types using the AI. Cavanagh et al. [6] and Menz et al. [11] proposed a quartile method and μ (mean) ± σ (standard deviation) method, respectively. The quartile method assumed that the distribution of AI data had a normal distribution, and the foot types were classified using the 1st and 3rd quartiles (Q1 and Q3) as reference values. The foot types were classified as concave when AI score was below Q1, normal when the AI was within the interquartile range (IQR, Q3–Q1), and flat foot when AI was over Q3 (3rd quartile). On the other hand, the μ ± σ method classifies the foot types using the mean and standard deviation of the AI data. The foot is a concave when AI is less than μ-σ, normal when AI is between μ − σ and μ + σ, and flat when AI is greater than μ + σ. Although these two methods utilize different criteria for the classification of foot types, the average values of AI and criteria points (Q1, Q3, μ − σ, μ + σ) are similar for concave feet and flat feet. Menz et al. showed that the μ ± σ method can yield a generalized distribution of AI data over the quartile method by using a larger amount of AI data when classifying foot types. Therefore, this study used the μ ± σ method for reliable abnormal foot classification. Figure 3a shows the labeled image data for concave, normal, and flat feet, and Figure 3b shows the distribution of the AI data collected in this study using the μ ± σ method. The foot types are classified as: concave (AI < 0.17), normal (0.17 ≤ AI ≤ 0.28), flat (AI > 0.28), which are almost the same as those obtained from Menz [11].

### 2.3. Pre-Processing of Image Data (Distribution of Pressure)

In the deep learning process, image data contain a lot of perceptual information, which makes them more valuable. However, the information in an image may cause overfitting owing to the excessive extraction of information, or may distort actual information owing to the indiscriminate combination and transformation of the original information. Therefore, an image preprocessing process is essential to efficiently utilize perceptual information. The classification model using raw data does not guarantee good accuracy; thus, there is the need for a pre-processing process that removes unnecessary parts which need not to be learned, and it is necessary to extract the ROI. In particular, because there are limitations with respect to the collection of sufficient data from experimental and clinical data, the preprocessing process takes a long time before using algorithms to achieve high accuracy [28,29,30]. Because the location and shape of the collected data varied owing to differences in the size, shape, and posture of each subject’s feet, the location and foot angle must be corrected to improve learning accuracy [29,30,31]. 

**Figure 4:**
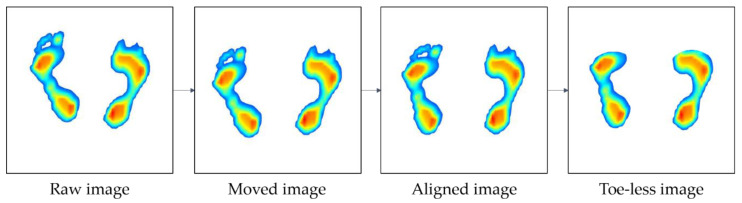
Image data pre-processing.

Thus, in order to improve the learning accuracy of the image data, the following pre-processing processes have been taken in this study. Figure 4 shows an overview of the image preprocessing process: (1) image positioning, (2) correction of foot angle, and (3) removal of the toe. As shown in Figure 4, the raw image is collected, after which it is moved to the center (image positioning). Next, the image is aligned to match the orientation of each foot (correction of foot angle), and finally, the toe is eliminated (removal of toe).

**Figure 5:**
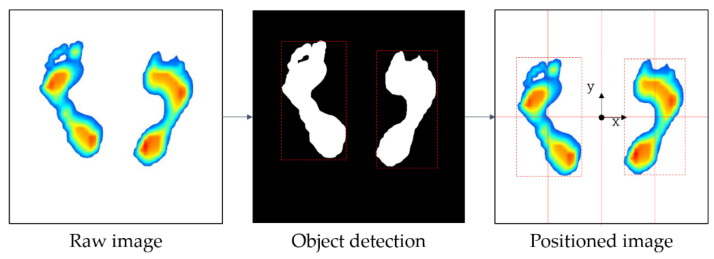
Image positioning method.

The image positioning, the first step in the image preprocessing process, was carried out through the following process. The foot pressure was measured at a specified standard location on the measuring device, but the measured foot positions were slightly different even in the same standard location because the foot shape, length, width, and standing positions were different. Therefore, all of the plantar data for 96 subjects were measured in different positions, and the subjects’ foot shapes and sizes were all different. Thus, if the original image data are used to generate learning models, it may degrade the performance of the models. Therefore, it is necessary to detect ROIs and place them in the center of the image for the right and left feet using data preprocessing. To do this, the color type of the image was changed from red, green, blue (RGB) to hue saturation value (HSV) because HSV has a narrower color range than RGB, making image detection easier. Subsequently, an object belonging to the color range (H: 0–120, S: 30–255, V: 30–255) is detected from the HSV color image, except for the white background. The detected left and right foot objects will be located in the center coordinates of each of the left and right images, divided by the y-axis of the raw image. Figure 5 shows the process of detecting and moving left and right foot objects based on raw image data. Once the left and right foot objects are detected using HSV, the coordinates of the rectangular area (red dashed line in Figure 5) surrounding the detected object (white area in Figure 5) are obtained. Finally, the left and right foot objects with the surrounding rectangular area move to the left and right centers of the raw image.

**Figure 6:**
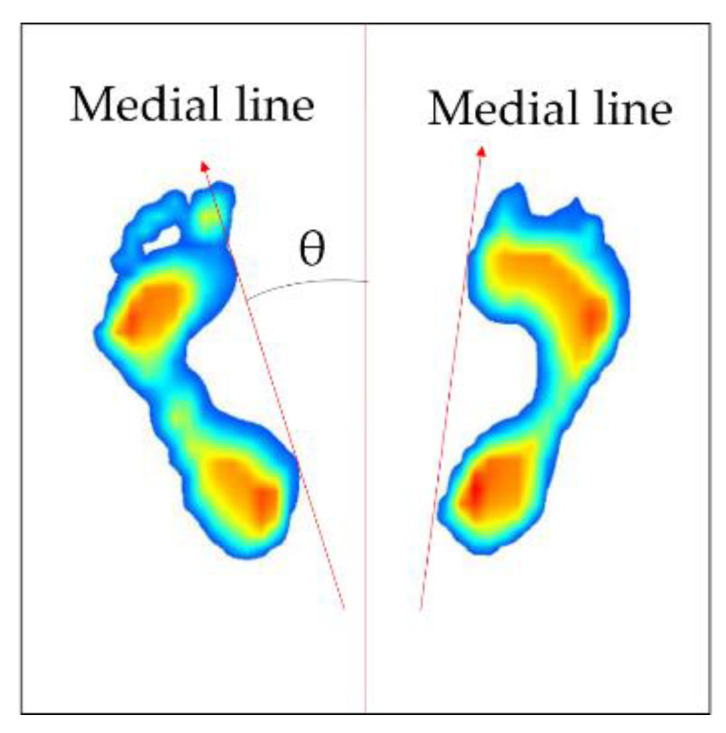
Definition of rotation angle.

Next, the process of correction of foot angle is conducted. The direction of each foot varies depending on the standing position; therefore, it is necessary to place them parallel to each other by rotating the direction of the positioned foot image in order to ensure they are in the same direction. To do this, the angle (θ) between the medial line of the foot and the vertical axis needs to be calculated first. Figure 6 shows how the rotation angle of the foot image is defined. The foot image is divided by the upper and lower parts at its center coordinates. Then, the inner line of the foot (red line) is defined by connecting the outermost points of each foot. Next, the foot image is rotated based on the center of each image using the rotation matrix in Equation (2) [29]. At this time, +θ is applied when rotating in a counterclockwise direction, and −θ is applied when rotating in a clockwise direction.

(2) $$R_{\theta }=\left[\begin{array}{cc} \cos\theta & -\sin\theta \\ \sin\theta & \cos\theta \end{array}\right]$$

**Figure 7:**
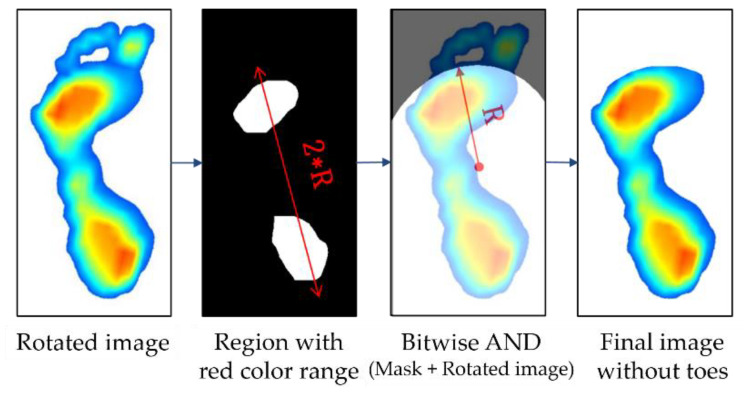
Steps in generating toe-less image.

Subsequently, the parts of toe on the image are eliminated. Because the AI score was calculated without using the area of the toe, the toe image is unnecessary. Thus, we removed the toe image from the foot image by employing the method proposed by Oliveira et al. [32]. Figure 7 shows the process of removal of the toe from an image whose angle is already corrected. The first step in the process of removing the toe image is to extract the region with the red color range (H: 0–40, S: 30–255, V: 30–255) from the image converted to HSV, which is similar to the object detection method used in the image data alignment. The region with the red color range corresponds to the area where the foot sole touches the ground and where the pressure is concentrated. Then, based on the center coordinates of the detected area, an arc with radius (R) is drawn to create a mask image, which will remain. At this time, R is defined as the distance between the heel ends from the center coordinates of the detection area. The final image is derived from the logical product (“AND”) of the original image and the mask image. Table 1 shows an example of a logical operation, where “True” refers to a color background, and “False” refers to a black background. 

After the position and angle of the image data are adjusted and unnecessary images are removed, normalization of the image’s color and data augmentation are needed to improve the accuracy of the classification model. As previously mentioned, image data, including RGB pixels, have a variety of colors; therefore, the use of raw data slows down the learning speed and falls into local optimization during the training process. To resolve this problem, normalization of image data was used to reduce the color variability of data in the preprocessing step before learning. In this study, image data were normalized using a contrast normalization method that unifies image brightness. Contrast normalization can reduce the difference in the brightness and environment between images and equalize them properly; therefore, it is a widely used image normalization method. The normalization process was carried out using Equation (3) by subtracting the average of the pixels ($X_{mean}$) from the pixels in the original image data (X), and then dividing the result by the standard deviation (*std*). While this is a simple image standardization method, it makes the features of images learning well known [33]. In the same way, the pressure value features were also pretreated using normalization.

(3) $$X’=\left(\frac{X-X_{mean}}{std}\right)$$

The quality of the data has improved through elaborated data preprocessing and normalization processes, but the number of data are still insufficient. In the learning process, the number of training data and unbalanced data can also greatly influence the performance of learning models. Thus, a sufficient number of image data need to be collected to obtain learning models with good performance. In general, it is known that a large number of abnormal cases and more than 60,000 sets of training data are needed to develop a high-performance deep learning algorithm using medical images [20]. However, it is very difficult to collect medical and clinical data because they often incur high test costs and take a long time. Data containing personal health information is highly sensitive and there is, therefore, a reluctance to disclose it, even for research purposes. Thus, the collected clinical data alone have limitations with respect to the development of high-performance deep learning models.

Many studies have attempted to augment image data in a variety of traditional ways such as image rotation, flip, shear and stretch [19]. The way to augment the image data is to create a transformed form of the image in a training dataset that belongs to the same class as the original image. Here, image transformation refers to the manipulation of images such as shifting, switching, and zooming, through which new images are created. In this study, the data were augmented by switching the left and right foot images, which were collected for 96 subjects. This is because the left and right feet typically have similar foot deformations and belong to the same foot deformation type, but there are still some variations that are suitable for use as augmented data. 

**Figure 8:**
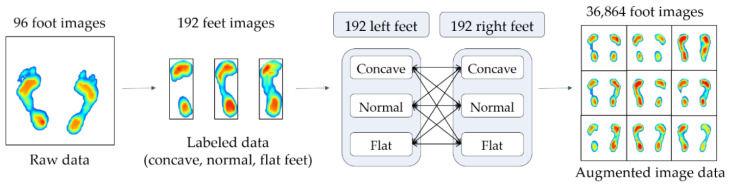
Data augmentation method.

Figure 8 shows the data augmentation process. First, the foot pressure data of 96 subjects are classified as concave, normal, and flat based on the AI, and the number of datasets are then doubled by switching both foot images, that is, 192 sets of data for the right and left feet with labels are now obtained. As shown in Figure 8, 192 datasets, divided into three groups for right and left feet, were mixed and increased to 36,864 (192 × 192) different sets of data for both feet. Accordingly, the three classes (concave, normal, and flat) were grouped into nine subclasses according to the AI scores. 

### 2.4. Pre-Processing of Numerical Data (Pressure Value)

Just as image data have been pre-processed, numerical data also requires pre-processing to improve the quality and accuracy of the data. The preprocessing of numerical data is carried out through the following processes: (a) feature extraction and selection, (b) normalization of selected features, (c) data augmentation. Since the process of normalizing and augmenting data in numerical data is the same as that in image data, only feature extraction and selection are described in detail in this section. 

**Figure 9:**
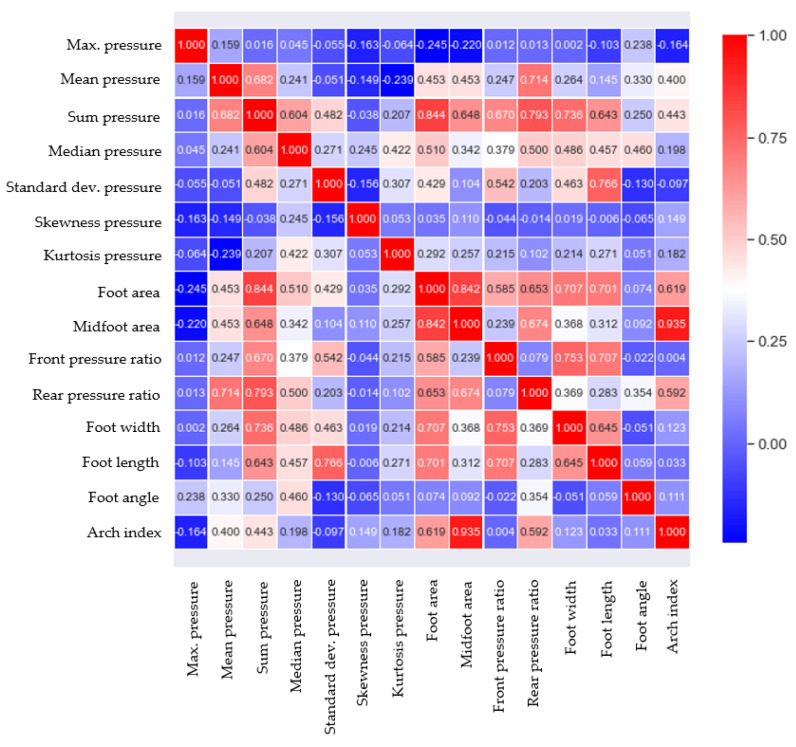
Correlation analysis results for features and arch index.

Feature analysis is an important process in determining the relationship between the features related to plantar pressure and foot deformation types, and by selecting the important features that determine foot deformation types, an accurate learning model can be developed. The main features used in plantar pressure analysis can be categorized into three types: statistical properties of pressure data, contact area-related parameters, and foot shape parameters [34,35,36,37]. In this study, 14 features, including three types of features, were extracted from the measured data for 96 subjects, as shown in Figure 2. When all of the extracted features are used as input variables in learning models, it can cause the redundancy of features, increased model complexity, and overfitting, and requires considerable computational power. Thus, a suitable number of features need to be determined. In this study, the Pearson coefficient was used to determine the strength of the linear association between 14 features and the AI. The correlation values of 0.2 or higher were chosen as the main features to increase the accuracy of the analysis, including various features that affect the AI, considering the complexity of the body. Accordingly, five features, namely the midfoot area, foot area, rear pressure ratio, sum pressure, and mean pressure, were selected and used for learning. Figure 9 illustrates the correlation analysis results obtained for the AI and 14 features, and Table 2 shows the selected features and their correlation coefficient values with AI.

## 3. Network Methodology

Two pre-processed heterogeneous types of data, namely image types and numerical types, need to be used to generate classification models for the foot deformation types. Section 3.1 and Section 3.2 introduce deep-learning models using fine tuning for image data of foot pressure and machine learning models for numerical data, respectively. Section 3.3 explains a stacking model that combines the machine-learning models and deep learning models introduced in Section 3.1 and Section 3.2 to further improve their robustness and accuracy. Section 3.4 describes how to evaluate the accuracy and robustness of learning models and the environment in which learning is performed. To generate learning models, the total dataset used was split: 80% for training and 20% for testing. The performance of the learning model was evaluated using the f1 score, as shown in Equation (4). The f1 score is defined as the harmonic mean of the precision and recall based on the confusion matrix in Table 3. The precision and recall are defined as the fraction of all positive predictions that are true positives, TP/(TP + false positive (FP)), and the fraction of all actual positives that are predicted positive, TP/(TP + false negative (FN)), respectively. The f1 score can provide a realistic measure of classification model’s performance by using both precision and recall, so that the F score is often used in evaluating the classifier performance [38].

(4) $$F1=\frac{2TP}{2TP+FP+FN}$$

### 3.1. Image Data Learning Based on Transfer Learning

#### 3.1.1. Algorithm of Image Data Learning Model

The CNNs can automatically extract learning features using convolution layers, and they are mainly used for learning text and image data types, which are features that are difficult to extract. Given these advantages, deep learning has seen remarkable growth and results in recent years. However, the deep learning needs to learn new data each time and to create a suitable neural network structure, which is very time consuming. Therefore, it is efficient to introduce well-trained and verified neural network structures and parameters using available source data, and to retrain them for a new set of target data to improve the learning performance. This process is called transfer learning. Transfer learning is considered as a CNN application method, and it is a well-known artificial intelligence methodology because it can improve model performance and reduce model generation time [39].

One of the basic requirements for transfer learning is that there should be a well-trained and validated model using source data. In the field of artificial intelligence, one of the best ways of selecting good models is to find models with a verified performance in image recognition competitions, such as the ImageNet Large Scale Visual Registration Challenge (ILSVRC). ILSVRC is an image recognition competition that accurately classifies datasets having 1000 categories and millions of image data. The deep-learning architecture that scored well in this competition is usually shared in public and is used in many studies [28,31,40,41]. The VGG16 and InceptionV3 models are used in this study; they are cited in many studies because of their good performance in the ILSVRC.

The learning time can be rapidly reduced if VGG16 and InceptionV3, which have good performance in image learning, are combined with fine-tuning. Fine-tuning, which is one of the training methods of transfer learning, performs fine weight updates at higher layers of the previously learned network to in order to obtain optimized models. This method is valuable when the size of the new data set is small [42], and the structures of the networks can be changed through fine-tuning [43]. For example, for our study, a pre-trained network classifies 1000 categories, but it can be adjusted at the output layer (softmax) to classify nine categories. The input image size is 224 × 224 × 3, which creates a classifier that classifies nine foot deformation shapes. Two networks have been implemented using the most popular deep learning frameworks, such as keras [44]. The fine-tuning strategies for VGG16 and InceptionV3 are described as follows.

The VGG16 model is composed of 16 layers and was released in 2014. To analyze the depth influence of the model, VGG16 confirmed the influence of the depth by stacking several layers of convolution layers with very small filters of 3 × 3 and deepening them. This has the effect of increasing the determinism of the decision function, and in 2014, it achieved an error rate of 7.32% with a slight difference in the object classification of the ILSVRC. Several studies use the VGG16 model because of its simplicity, fast epoch, and ease of understanding and transformation [45].

#### 3.1.2. Fine-Tuning Strategy for Ensemble Models

This study used a fine-tuning strategy that employs VGG16 pre-trained using ImageNet datasets. Up until block 4 of the VGG16, the weights learned by ImageNet datasets are frozen, and from block 5, the weights of the convolution layer, the last three fully connected (FC) layers and output layer (softmax layer) are fine tuned. At this time, the number of FC nodes was adjusted to 1000, and a drop-out layer (rate = 0.6) was added to prevent overfitting. The input image size is 224 × 224 × 3, which creates a classifier for nine foot shapes. Then, the optimizer that updated the weight parameters used root mean square propagation (RMSprop) (learning rate = 10 × 10^−5^). This is to prevent large changes in shared weights and to optimize new data sets [42].

InceptionV3 is an image classification model that is often used as a pre-trained model for fine-tuning learning. It is the 3rd version in a series of deep learning convolution architectures, which began with InceptionV1, and it was trained on more than one million images obtained from the ImageNet database. It is generally known that when learning large data, the deeper the layer and the wider the network, the better the performance. However, if the layer becomes deep, the gradient value disappears (vanishing gradient), and if the network becomes wide, the number of parameters increases, causing increased computational time and overfitting, and making learning very difficult. To compensate for this shortcoming, InceptionV1 introduced a subnetwork, the Inception module, to reduce the number of parameters that are finally learned by reducing the connection between nodes [41]. Subsequently, through improvements of the inception module up until InceptionV3, it exhibited an accuracy of more than 78.1% in the ImageNet dataset [46].

To fine-tune the InceptionV3 models pre-trained by ImageNet, the entire Inception module was fine-tuned. Similar to the VGG16’s fine-tuning strategy, the dropout ratio is 0.6, and the number of fully connected neurons is 1024. The optimizer that was employed to update the weight parameter used RMSprop (learning rate = 10 × 10^−5^) because it should be lower than the traditional learning rate to prevent overfitting.

### 3.2. Numerical Data Learning Based on Features

Using the five features extracted from the foot pressure data described in Section 2.4, in this study, nine types of foot deformation types were classified using k-NN and CART [26]. Both methods are commonly used as classification models because they have simple structures that are easy to understand and have high performance. The parameters used in both models were optimized such that they maximized the performance of the learning models through hyperparameter tuning using the Bayesian method [47].

k-NN is a non-parametric classifier that is based on supervised learning, and it has been used as a reference classifier in many pattern classification problems. k-NN is a popular classification algorithm owing to its simplicity and ease of implementation, and it is thus widely used in classification problems in many fields. k-NN stores all available data and classifies new data based on a similarity measure, such as distance functions. The k−NN directly calculates the distance between the new data and *k* sets of training data to identify the nearest neighbors of the new data. The *k* data groups are formed in ascending order of the measured distance, and the new data fall into one class with the highest frequency among the classes to which the surrounding *k* sets of data belong.

It is necessary for the type of distance function and the *k* value to be determined before performing k−NN. In general, the Euclidean distance is the most widely used method when calculating the distance between two points, and numeric and categorical data sets are known to be suitable for Euclidean distance-based k−NN [48]. A higher *k* can produce a model that is more insensitive to outliers (harder), but it requires a large number of computations. In this study, the *k* value was optimized using Bayesian optimization; therefore, the performance of the model was maximized, and the calculation time was not significantly long. The range of *k* was chosen as [1,1000] considering the dataset size, and the f1 score according to *k* showed the highest performance with an f1 score of 0.9 when *k* was 7; therefore, the *k* value was set to 7 when generating the k−NN model.

(5) $$d=\sqrt{\sum_{i=1}^{k}{\left(x_{i}-y_{i}\right)}^{2}}$$

CART is a non-parametric method of generating a decision tree by recursively splitting the data into partitions according to decision rules, and it is one of the most well−known methods of decision tree methodology. In addition, because it is intuitive and easy to handle, it is used as a basic model for machine-learning algorithms in many fields because it allows researchers to easily explain the analysis results [25].

CART is an algorithm that performs binary separation using the Gini index for categorical data. The Gini index is a measure of how often randomly chosen data set would be incorrectly identified, and it is also called the Gini impurity measure because purity decreases when there are heterogeneous data in the same group, while impurity or uncertainty increases. Equation (6) shows how to calculate the Gini index, where $p_{i,k}$ represents the percentage of samples in class *k* among the training samples on the *i*th node for binary separation. If all data belong to a single class, *G_i_* becomes zero, i.e., the binary separation is performed well. If all data are randomly distributed across various classes, *G_i_* becomes one, which means that the binary separation is performed incorrectly.

(6) $$G_{i}=1-\overset{n}{\underset{k=1}{\sum }}p_{i,k}^{2}$$

The CART algorithm first divides a training set into two subsets using a single feature *k* and a threshold *t_k_*. The *k* and *t_k_* values are selected such that the training data set should be divided into the purest subsets having homogeneous data within the same class. To do this, the following cost function should be minimized:

(7) $$J\left(k,t_{k}\right)=\frac{m_{left}}{m}G_{left}+\frac{m_{right}}{m}G_{right}$$

where *G_left_* and *G_right_* are the *G_i_* values of the left and right subsets, respectively. *m* is the total number of samples, and *m_left_* and *m_right_* are the number of samples for the left and right subsets, respectively. The CART algorithm is performed by continuously pruning in the direction of minimizing the cost function of the decision tree. This process stops when it reaches max_depth (maximum depth) or stops when it cannot find a split that reduces the impurity.

CART has few restrictions on training data; therefore, it attempts to fit it as close as possible to the training data, resulting in the generation of a complex learning model. Thus, to control the complexity of the CART model, the maximum depth related to model complexity was optimized using Bayesian optimization in the same way as k−NN. As a result of the hyperparameter optimization, the f1 score was 0.86 when the maximum depth value was 9 within a range of [0,10]; therefore, the maximum depth value was selected as 9.

### 3.3. Stacking Ensemble with Cross-Validation

Ensemble learning has been used in recent years to improve the weak performance of the single learning model and to solve data bias problems [49]. The stacking ensemble is an ensemble learning method that combines different types of base learning models to create a new model. Stacking ensemble learning builds up metadata by stacking the predicted values of the base models and derives the final predictive values using meta learners [50,51].

**Figure 10:**
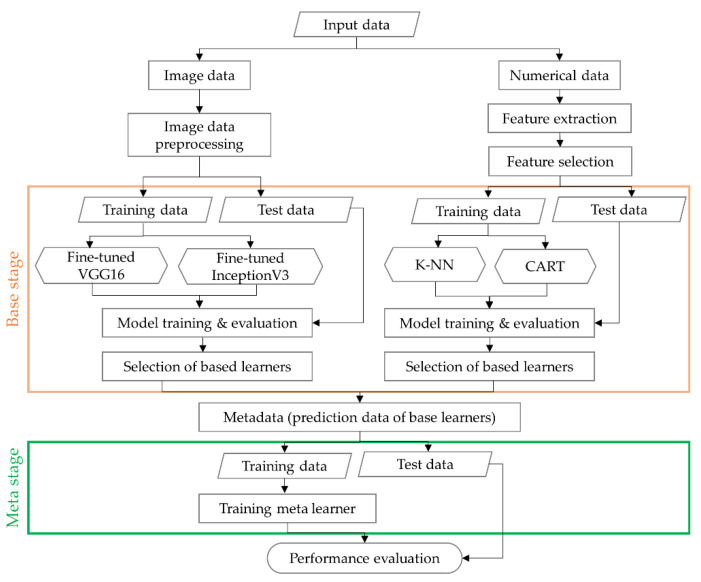
Scheme showing the proposed stacking ensemble model.

In Section 3.1 and Section 3.2, the accuracy of the classification learning models that were generated for image data and numerical data was compared, and the VGG16 and k-NN models with the highest performance for each data type were selected as the base classifiers. The prediction data from two base classifiers are generated from the layer composed of the basic classifiers to form the metadata, which are used as the input to the stacking ensemble learning, as shown in Figure 10. In order to prevent the same data from being learned when learning the prediction values for the basic classification model, a cross-validation method is applied. To obtain the final prediction data, the prediction data of the base models, i.e., metadata that were produced using five cross-validation methods, were used as input data for meta classifiers that have a shallow neural network including one layer with 1024 nodes and 0.3 of dropout rate.

### 3.4. Evaluation Method and Hardware for Learning Model

To evaluate the proposed model, stratified k-fold cross-validation was performed. The k-fold cross validation method is often used for model evaluation as it can enable us to avoid overfitting training datasets and improve model generalization. The k-fold cross validation divides the training dataset equally into *k* groups (called folds) and selects (*k* – 1) test folds and 1 validation fold. Then, a total of *k* validations were performed using different test folds for each validation. However, if the distribution of classes in the data set is unbalanced, when constructing the subset, there is data bias in the subset, resulting in a fatal error in model validation. Because the dataset used in this study has class imbalances, subsets were generated using stratified cross-validation according to the data distribution [52], which can preserve the percentage of data for each class. The average f1 score of the subset was employed to evaluate the model’s performance using stratified 5-fold cross-validation. The learning environment for training and testing is based on Python 3.7 on PCs running Windows 10 with a 64-bit operating system and an x64-based processor, Intel (R) Xeon (R) CPU E5−2650 v4 @ 2.20 GHz processor, 8 GB RAM, and an Intel Xeon v4 processor. It was performed using a tensorflow2.0 GPU (GeForce GTX 1070) and the cuda compilation tool (Release 10.0, V10.0).

## 4. Result and Discussion

**Figure 11:**
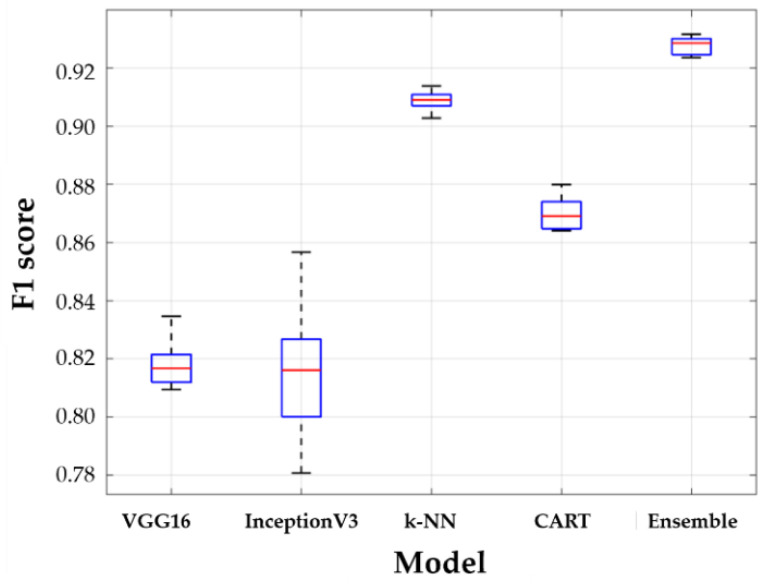
Results of learning models.

The entire dataset was divided as follows: 80% for the training dataset and 20% for the test data set. The training dataset was used for model training based on stratified 5-fold cross-validation. Finally, each model that was trained using each subset was evaluated for a test dataset by calculating the average of the f1 scores. Figure 11 and Table 4 show the performance results obtained for all models. The boxplots in Figure 11 show the distribution of five f1 cores by 5-fold cross validation. In Figure 11, the horizontal lines above and below the box are the first and third quartiles, respectively, the center lines are the median values (second quartiles), and the outermost horizontal lines along the dashed lines represent the maximum and maximum values of the f1 score, respectively. The higher the accuracy of the model, the closer the box is to 1, and the higher the robustness of the model, the narrower the gap between the outermost values. Table 4 shows the numerical results of statistical parameters such as mean, standard deviation, median, min and max for five f1 scores.

When comparing the f1 scores, the VGG16 model has a similar accuracy compared to the InceptionV3 model performance, its variance is smaller than that of InceptionV3. InceptionV3 uses a deep network to extract the features of the image data, but the data used in this study are often at the boundary of the criteria for the classification of foot deformation types, which is somewhat ambiguous in features, resulting in poor performance compared to VGG16. The k-NN and CART using the numerical data only, k-NN has a better performance than CART. By using algorithms that determine categories based on a majority of *k* most similar data, k-NN is insensitive to anomalies and has the lowest deviation in the results, while CART uses the optimized maximum depth values to prevent overfitting; therefore, k-NN performs better in this study. However, because the stacking ensemble model can achieve the advantages of two different models for both image data and numerical data, the stacking ensemble model trained with metadata shows the best performance with the highest accuracy and the smallest variance. Therefore, it can be seen that an ensemble model is essential to increase the classification performance of a foot deformation model.

Models that are trained with image data (VGG16 and InceptionV3) show relatively lower performance than models (k-NN and CART) trained on numerical data. This is because it is more difficult to extract features in the process of extracting plantar image data than numerical data. Table 5 shows the extracted plantar pressure image and its AI scores and foot deformation types. The principle of CNN, which is the basis of the model of VGG16 and InceptionV3, is to extract features by moving the kernel within the image. However, the images at the boundary of the foot are similar in color and shape; therefore, the features are extracted and learned in the same group. However, the error rate of classification is high because they are actually different types of images. In the histogram of the AI scores (Figure 3b), the data in the range of ± 0.1 of the boundary is 38 out of 192, which is approximately 19.79% of the total. A considerable amount of data are distributed near the boundaries of the criteria for classification, and images near the boundaries are likely to produce errors with respect to foot-type classification because similar characteristics are extracted. However, in areas other than the boundaries of the foot classification criteria, both VGG16 and InceptionV3 have high classification performance for foot deformation types.

## 5. Conclusions

This study proposed a data-driven artificial intelligence methodology to diagnose foot health using plantar pressure data. To do this, a plantar pressure measurement device was used, and image data (pressure distribution) and numerical data (pressure value) were first collected, after which data preprocessing methods for two heterogeneous types of data were developed and performed. Second, classification models of foot deformation types for image data and numerical data, such as VGG16, InceptionV3, k-NN and CART were generated. Third, in order to improve the robustness and accuracy of the model, metadata were formed based on the predicted values of each model. A stacking ensemble combining VGG16 and k-NN, which have a better performance compared with the other models, was proposed. The stacking ensemble model combines the advantages of two heterogeneous models to improve the accuracy and robustness of other models that are used alone.

The proposed classification model for determining the foot deformation type obtained accuracy with f1 score of 92.55% using only pressure data measured on 96 subjects through elaborated data pre-processing and artificial intelligence model generation. The proposed model achieved high classification accuracy with only a small amount of data, but if the number of foot pressure data sets is increased and the diagnosis results of experienced medical personnel are added, it is expected that a more accurate foot health diagnostic model can be developed. The developed model is considered to be useful as an objective tool for medical personnel to diagnose foot health, and can be extended to diagnostic models of various medical data.

## Figures and Tables

**Table 1:** Logical product.

A	B	Result
False	False	False
False	True	False
True	False	False
True	True	True

**Table 2:** Selected features.

Feature	Correlation Coefficient
Midfoot area	0.935
Foot area	0.619
Rear pressure ratio	0.592
Sum pressure	0.443
Mean pressure	0.400

**Table 3:** Structure of the confusion matrix.

	Actual Positive	Actual Negative
**Predicted Positive**	True positive (TP)	False positive (FP)
**Predicted Negative**	False negative (FN)	True negative (TN)

**Table 4:** Descriptive statistics of model f1 score in 5-folding cross-validation.

	Fine-Tuned VGG16	Fine-Tuned InceptionV3	k−NN	CART	Stacking Ensemble
Mean	0.8181	0.8153	0.9088	0.8700	0.9255
Std.	0.0097	0.0274	0.0039	0.0064	0.0042
Min	0.8094	0.7807	0.9028	0.8640	0.9188
Median	0.8168	0.8161	0.9090	0.8691	0.9259
Max	0.8346	0.8567	0.9139	0.8800	0.9304
Computational time [min.]	200	240	10	2	213

**Table 5:** Image of arch index score’s labeling boundary.

Image	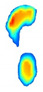	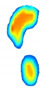	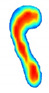	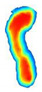
**AI score**	0.168	0.171	0.279	0.281
**Labeling type**	Concave	Normal	Normal	Flat
